# Unusual evolution of lipoid pneumonia on CT

**DOI:** 10.36416/1806-3756/e20260032

**Published:** 2026-05-22

**Authors:** Flávia Angélica Ferreira Francisco, Gláucia Zanetti, Edson Marchiori

**Affiliations:** 1. Universidade Federal do Rio de Janeiro, Rio de Janeiro (RJ) Brasil.

**Figure 1:**
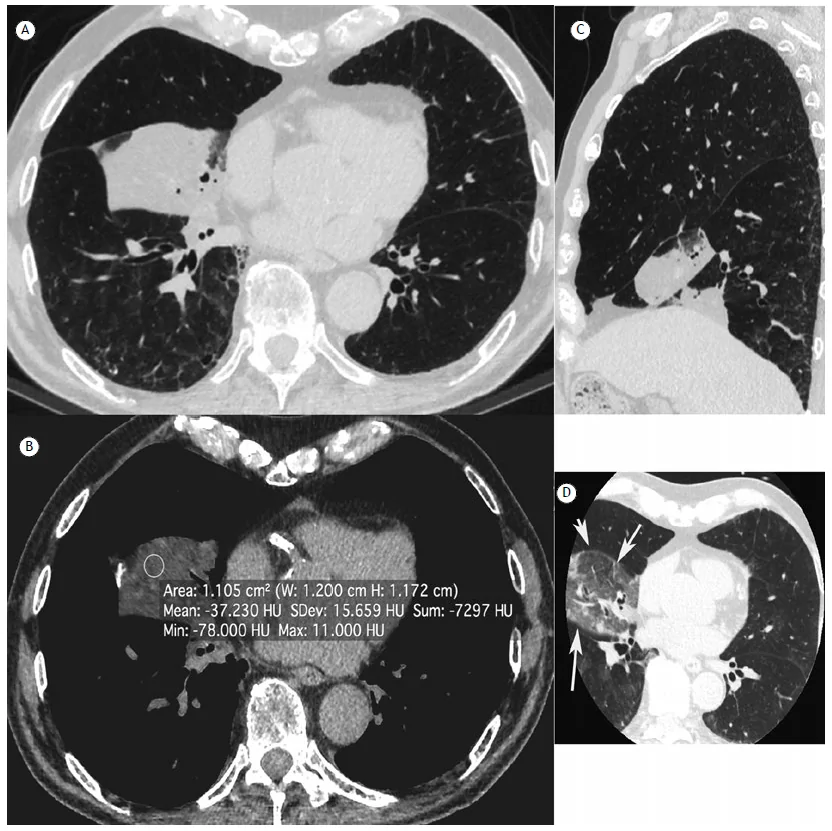
Chest CT images with axial (A) and sagittal (B) reconstruction showing consolidations in the middle lobe and the anterior basal segment of the right lower lobe. In (C), the middle lobe consolidation demonstrates negative attenuation (−37 Hounsfield units). In (D), an axial CT examination performed 2 years previously for coronary evaluation shows rounded ground-glass opacity (arrows) at the site of the subsequent middle lobe consolidation.

An 84-year-old man presented with a 3-year history of dyspnea and cough, sometimes productive, with clear expectoration. Laboratory test results were within normal limits. Chest radiography demonstrated an area of consolidation in the middle lobe. Chest CT revealed heterogeneous consolidations in this region, with internal areas of negative attenuation (-37 Hounsfield units; Figures 1A and 1B). The patient had used mineral oil for the treatment of chronic constipation, but had discontinued its use 3 years previously. He had undergone a coronary CT examination 2 years previously, which demonstrated a heterogeneous consolidation with ground-glass opacities and areas of consolidation in the middle lobe (Figure 1D). The patient’s clinical history and tomographic findings were considered to be characteristic of lipoid pneumonia (LP), obviating the need for BAL or lung biopsy.

LP is an uncommon disease caused by the intra-alveolar accumulation of aspirated or inhaled lipids and lipid-laden macrophages. It can be classified as endogenous or exogenous. The diagnosis of exogenous LP is based on a history of mineral oil aspiration, radiological findings (in particular, foci of fat attenuation within areas of consolidation on CT) and/or the presence of lipids in BAL fluid or lung biopsy specimens.^1^ ^-^ ^4^ The likely explanation for the evolution of the ground-glass opacities into consolidation on follow-up examination in this case is that the lipid material had continued to trigger a local inflammatory reaction despite the patient’s discontinuation of mineral oil use before the first examination.
